# Intestinal Microbiota in Colorectal Adenoma-Carcinoma Sequence

**DOI:** 10.3389/fmed.2022.888340

**Published:** 2022-07-20

**Authors:** Hanju Hua, Yanhong Sun, Xinjue He, Yi Chen, Lisong Teng, Chao Lu

**Affiliations:** ^1^Department of Colorectal Surgery, The First Affiliated Hospital, Zhejiang University, Hangzhou, China; ^2^National Clinical Research Center for Child Health, National Children’s Regional Medical Center, The Children’s Hospital, Zhejiang University School of Medicine, Hangzhou, China; ^3^Department of Gastroenterology, The First Affiliated Hospital, Zhejiang University School of Medicine, Hangzhou, China; ^4^Department of Surgical Oncology, The First Affiliated Hospital, Zhejiang University School of Medicine, Hangzhou, China

**Keywords:** intestinal microbiota, colorectal cancer, adenoma-carcinoma sequence, colorectal adenoma, 16S rRNA

## Abstract

**Background:**

Most colorectal cancer (CRC) cases are sporadic and develop along the adenoma-carcinoma sequence. Intestinal microbial dysbiosis is involved in the development of colorectal cancer. However, there are still no absolute markers predicting the progression from adenoma to carcinoma. This study aimed to investigate the characteristics of intestinal microbiota in patients with colorectal adenoma and carcinoma and its correlations with clinical characteristics.

**Methods:**

Fecal samples were collected from 154 patients with CRC, 20 patients with colorectal adenoma (AD) and 199 healthy controls. To analyze the differences in the intestinal microbiota, 16S rRNA gene sequencing was conducted.

**Results:**

At the genus level, there were four significantly different genera among the three groups, namely Acidaminococcus, Alloprevotella, Mycoplasma, and Sphingobacterium, while Acidaminococcus significantly decreased with the order of Control-AD-CRC (*P* < 0.05). In addition, Parvimonas, Peptostreptococcus, Prevotella, Butyricimonas, Alistipes, and Odoribacter were the key genera in the network of colorectal adenoma/carcinoma-associated bacteria. The top 10 most important species, including *Butyricimonas synergistica*, *Agrobacterium larrymoorei*, *Bacteroides plebeius*, *Lachnospiraceae bacterium feline oral taxon 001*, *Clostridium scindens*, *Prevotella heparinolytica, bacterium LD2013*, *Streptococcus mutans*, *Lachnospiraceae bacterium 19gly4*, and *Eubacterium hallii*, showed the best performance in distinguishing AD from CRC (AUC = 85.54%, 95% CI: 78.83–92.25%). The clinicopathologic features, including age, gender, tumor location, differentiation degree, and TNM stage, were identified to be closely linked to the intestinal microbiome in CRC.

**Conclusion:**

Several intestinal bacteria changed along the adenoma-carcinoma sequence and might be the potential markers for the diagnosis and treatment of colorectal adenoma/carcinoma. Intestinal microbiota characteristics in CRC should account for the host factors.

## Introduction

Colorectal cancer (CRC) is the fourth most deadly cancer worldwide (2). Due to the popularization of physical examination, the incidence and mortality of CRC in individuals over 50 years old have steadily decreased, while the incidence under the age of 50 has been on the rise (3). More than half of sporadic CRCs arise *via* adenoma-carcinoma sequence, starting as premalignant lesions represented by conventional, tubular, or tubulovillous adenomas (4). Therefore, understanding the characteristics of adenoma-carcinoma sequence is critical for CRC prevention. The pathogenesis of CRC is related to multiple factors, including diet, lifestyle, environmental factors, genetic mutation, and so on (5, 6). In recent years, an increasing number of studies have shown that intestinal microbiota play important roles in the development of CRC (7). Chen et al. reported that patients with CRC have shown reduced bacterial richness and diversity compared with those of healthy controls (8). According to metagenome-wide association study, Feng et al. found that fecal microbiome-based strategies may be propitious to the early diagnosis of CRC (9). It is reported that *Streptococcus bovis*, *Fusobacterium nucleatum*, and *Enterococcus faecalis* are closely related to the pathogenesis of CRC (10). In addition, *Escherichia coli*, as a class of symbiotic bacteria, has been reported higher levels of colonic colonization in CRC (11). On the other hand, some bacteria, such as *S. thermophilus* and *Clostridium butyicum* are depleted in CRC patients, which suggest that these bacteria may exert a protective effect against CRC (10). The results of the above studies show that there is currently no consistent result about intestinal microbiota and CRC. The relationship between the two is called “Alpha-Bug” (12) or “Driver-Passenger Model” (13) by researchers. Microbiota-associated mechanism in CRC carcinogenesis is highly complex, including biofilm, genotoxins, pathogenic bacteria, inflammation, metabolites, and oxidative stress (14).

Certain factors may affect the consistency of results in studies of the effect of gut microbiota on CRC, including the differences in samples (fecal or mucosal tissue), processing method (RT-PCR or high-throughput analyses), different stage of tumor or location differences between the left- and right-side (15). Therefore, there is controversy on markers predicting the progression of adenoma-carcinoma sequence.

In this study, the first purpose was to characterize the microbiota differences along the adenoma-carcinoma sequence based on 16s rRNA gene sequencing. The second aim was to explore the correlations between the intestinal microbiota and its clinical characteristics. Whether clinicopathologic features, including age, gender, tumor location, differentiation degree, and tumor node metastasis (TNM) stage, were closely linked to the intestinal microbiome in CRC. Based on the adenoma-carcinoma sequence, our findings provide more knowledge for elucidating the tumorigenesis and offer a basis for the development of more effective strategies for the clinical treatment of CRC in the future.

## Materials and Methods

### Study Population and Sample

Participants were outpatients who received colonoscopies by experienced endoscopists in our hospital. The exclusion criteria included: (1) patients older than 90 years of age; (2) patients with a personal history of colorectal cancer, colorectal adenoma, inflammatory bowel disease (colitis-associated pathway is another different molecular pathways), or a family history of colorectal cancer (potential genetic confounding factors); (3) patients had used antibiotics within 2 months or received chemotherapy/radiation treatments within 6 months; (4) individuals who like red and processed meat. Informed consent was obtained from the participants and their privacy was respected. Fresh fecal samples (≥ 1 g) were collected from all participants before colonoscopy and immediately frozen at -80°C until further processing for 16S rRNA gene sequencing analysis. The clinical characteristics of the patients were recorded, including age, sex, tumor size, location, TNM stage, differentiation degree, pathological pattern, and histology. In addition, there were no geographic differences among the patients included in the study, so there was no difference in dietary patterns. Types of adenomas followed the ICD10 and histological classification.

The study protocol was approved by the Medical Ethics Committee of the First Affiliated Hospital, Zhejiang University School of Medicine (2017-869).

### DNA Extraction and 16s rRNA Gene Sequencing

Total genomic DNA extraction from fecal samples was performed using QIAamp DNA Stool Mini Kit (QIAGEN, Germany) following the manufacturer’s instructions. The quality and concentration of DNA were verified with 2% agarose gel (Tanon, China) and NanoDrop spectrophotometer (Thermo Fisher Scientific, Waltham, MA, United States).

High-throughput Illumina sequencing of the V3–V4 variable regions of the 16S rRNA gene was performed using universal primers 343F (5′-TACGGRAGGCAGCAG-3′) and 798R (5′- AGGGTATCTAATCCT-3′). The PCR reaction was carried out with 15 μL of Phusion High-Fidelity PCR Master Mix (New England Biolabs), 0.2 μM of forward and reverse primers, and about 10 ng template DNA. Thermal cycling consisted of initial denaturation at 98°C for 1 min, followed by 30 cycles of denaturation at 98°C for 10 s, annealing at 50°C for 30 s, and elongation at 72°C for 30 s followed by a final extension of 5 min at 72°C.

The amplicon was purified with AMPure XP beads (Beckman Coulter, Brea, CA, United States) and subjected to a secondary PCR reaction. The final amplicon was quantified using a Qubit dsDNA assay Kit (Life Technologies, Carlsbad, CA, United States). Sequencing library was generated using TruSeq^®^ DNA PCR-Free Sample Preparation Kit (Illumina, San Diego, CA, United States) following the manufacturer’s recommendations and assessed on the Qubit^®^ 2.0 Fluorometer (Thermo Fisher Scientific, Waltham, MA, United States) and Agilent Bioanalyzer 2100 system. The library was sequenced on an Illumina NovaSeq platform and 250 bp paired-end reads were generated.

### Bioinformatics and Statistical Analysis

The FASTQ files were processed using QIIME (V1.9.1^1^). Paired-end reads were merged using FLASH (V1.2.7^2^). Quality filtering on the raw tags was performed under specific filtering conditions to obtain high-quality clean tags. The effective tags were obtained by detecting chimera sequences and removing the chimera sequences using UCHIME algorithm (UCHIME Algorithm^3^) based on the reference database (Silva database^4^). Sequences with 97% sequence homology were assigned to the same operational taxonomic units (OTUs) by Uparse software (version 7.0.1001^5^). The representative sequence for each OTU was screened for further annotation. All representative reads were annotated and blasted against the SILVA database (version 123, see footnote 4) using RDP classifier (confidence threshold was 70%). OTUs abundance information was normalized using a standard of sequence number corresponding to the sample with the least sequences.

Alpha diversity and beta diversity analysis were performed by QIIME (version 1.9.1). Alpha diversity was applied for analyzing complexity of species diversity for samples. The community richness and diversity were described by the Chao index and Shannon index, respectively. Beta diversity analysis was used to evaluate comparisons between groups among samples and assessed by non-metric multidimensional scaling (NMDS) analysis and principal coordinates analysis (PCoA) by using weighted UniFrac distance matrix. Linear discriminant analysis (LDA) effect size (16) was used to identify differentially abundant bacterial taxa associated with groups of participants. The LDA value threshold was set at 4. *T*-test and Wilcox test were employed to assess the significance by QIIME (version 1.9.0).

## Results

### Characteristics of Subjects

A total of 373 participants, including 154 patients with CRC (mean age 65.81 ± 10.98 years old, 63.64% male), 20 patients with AD (mean age 64.35 ± 12.89 years old, 75.00% male) and 199 healthy controls (mean age 59.48 ± 3.20 years old, 58.29% male) were enrolled in this study. The control group was younger, but we found the same conclusion after adjusting for age as a confounder. The clinical characteristics of the patients were shown in Table 1. The mean size of carcinoma was 4.55 ± 2.18 mm and most of the tumors were found in the left colon (75.97%). In the CRC group, the differentiation degree concentrated in the moderate degree (75.32%) and mainly tubular adenocarcinoma (85.06%).

**TABLE 1:** Demographic and clinical characteristics of patients and controls.

Characteristics	Control	AD	CRC
Number	199	20	154
Age, years	59.48 ± 3.20	64.35 ± 12.89	65.81 ± 10.98
Sex			
Male	116 (58.29%)	15 (75%)	98 (63.64%)
Female	83 (41.71%)	5 (25%)	56 (36.36%)
Location*^a^*			
Left	–	19 (95%)	117 (75.97%)
Right	–	1 (5%)	37 (24.03%)
Size, mm	–	–	4.55 ± 2.18
TNM stage*^b^*			
I	–	–	33
II	–	–	51
III	–	–	43
IV	–	–	10
NA	–	–	17
Differentiation degree			
Poor	–	–	9
Moderate	–	–	116
Well	–	–	9
NA	–	–	20
Pathological pattern			
Protrude	–	–	63
Ulcerative	–	–	69
NA	–	–	22
Histology			
Tubular	–	–	131
Mucinous	–	–	20
NA	–	–	3

*CRC, colorectal cancer; AD, adenomas.*

*^a^The left colon was defined as the rectum, sigmoid, and descending colon; the right colon was defined as the transverse colon, ascending colon, and cecum.*

*^b^Tumor node metastasis (TNM) stage.*

### Summarization of 16S rRNA Gene Sequencing Results

**FIGURE 1:**
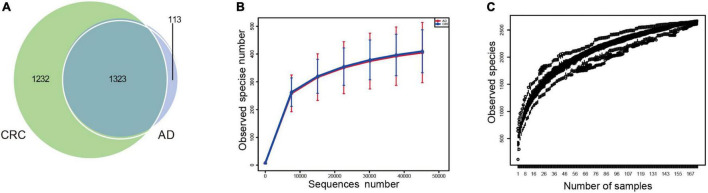
**(A)** Venn diagram illustrating the total, unique, and shared number of OTUs predicted for AD and CRC group datasets; **(B)** Rarefaction curve of OTU; **(C)** Species accumulation boxplot.

**FIGURE 2:**
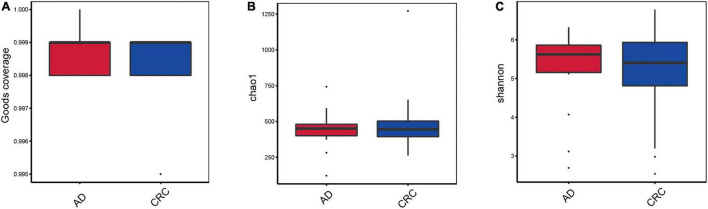
**(A)** Box plot based on Good’s coverage; **(B)** Alpha diversity analysis based on the Chao1 index in the AD and CRC group (*P* = 0.95); **(C)** Alpha diversity analysis based on the Shannon index in the AD and CRC group (*P* = 0.74).

A total of 2,668 OTUs were generated from 164 patients’ samples. A Venn diagram showed that 1,323 OTUs were shared between AD and CRC, while 113 unique OTUs existed in AD, and 1,232 unique OTUs existed in CRC (Figure 1A). Rarefaction curve and species accumulation boxplot were shown in Figures 1B,C. Moreover, the value of Good’s coverage for each group was over 99.8% (Figure 2A). No significant difference in alpha diversity was observed between the AD and CRC group (*P* = 0.95 for Chao index, Figure 2B; *P* = 0.74 for Shannon index, Figure 2C).

**FIGURE 3:**
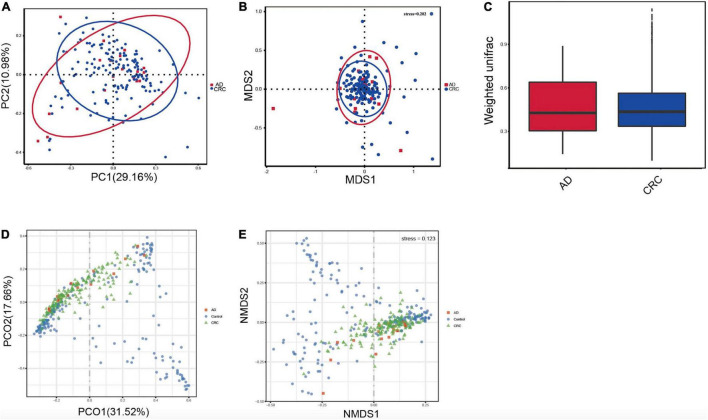
**(A)** Principal coordinates analysis (PCoA) based on weighted unifrac distance matrix in the AD and CRC group [Pr (> F) = 0.33]; **(B)** Non-metric multidimensional scaling (NMDS) based on weighted unifrac distance matrix in the AD and CRC group (stress = 0.202); **(C)** Beta diversity analysis by weighted UniFrac distance in the AD and CRC group (*P* = 0.88); **(D)** Principal coordinates analysis (PCoA) based on weighted unifrac distance matrix among Control, AD, and CRC group; **(E)** Non-metric multidimensional scaling (NMDS) based on weighted unifrac distance matrix among Control, AD, and CRC group (stress = 0.123).

A mild separation between AD and CRC was observed and no significant difference in microbiota composition was detected by PCoA analysis [Pr (> F) = 0.33 for weighted UniFrac distance, Figure 3A]. NMDS analysis also revealed that the microbiota composition between two groups was similar, and samples from AD and CRC mostly overlapped with one another (stress = 0.202 > 0.2, Figure 3B). The analysis of beta diversity revealed that fecal microbial communities between AD and CRC were not distinct from each other (*P* = 0.88 for weighted UniFrac distance, Figure 3C). However, there was more distribution in the control samples than that in AD or CRC samples both in the PCoA and NMDS plots (stress = 0.123 < 0.2, Figures 3D,E), suggesting that the fecal microbial communities between the Control and the AD or CRC group were different.

### Specific Bacterial Taxa Associated With Adenoma-Carcinoma Sequence

**FIGURE 4:**
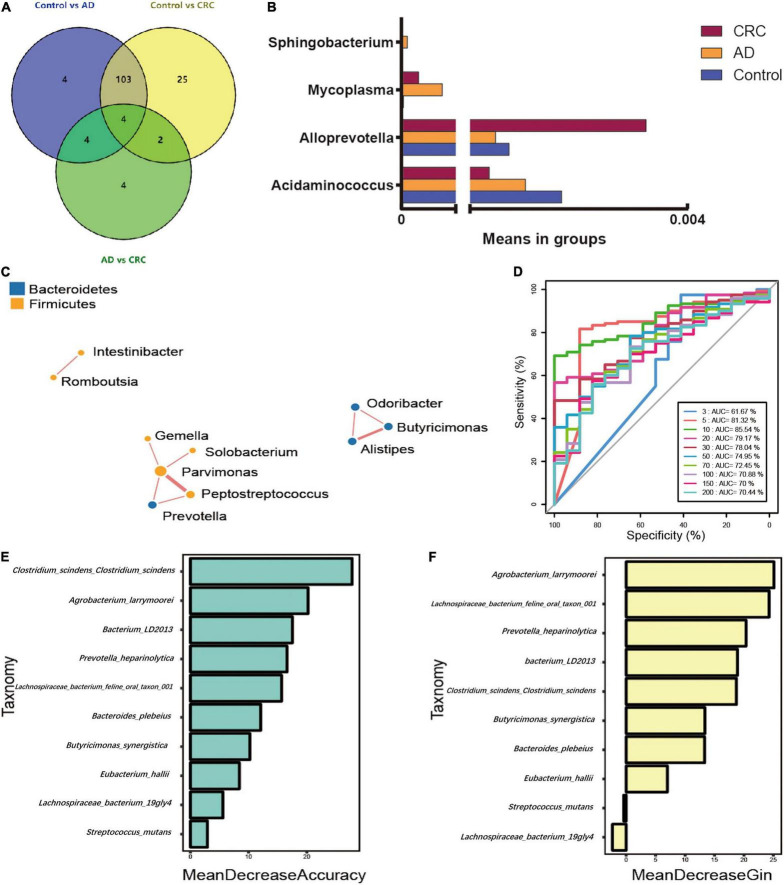
**(A)** Venn diagram illustrating the total, unique, and shared number of significant pairwise difference at the genus level among the Control, AD, and CRC groups; **(B)** The relative abundance of the significant changed genera among the three groups; **(C)** Microbial association network for colorectal adenoma/carcinoma-associated genera; **(D)** Receiver operating curve (ROC) by Random Forest analysis for distinguishing AD patients from CRC; **(E)** Mean Decrease Accuracy (MDA) coefficients of the 10 most important species in distinguishing AD patients from CRC; **(F)** Mean Decrease Gini (MDG) coefficients of the 10 most important species in distinguishing patients with AD from patients with CRC.

The significant analysis based on Wilcox test showed that 115 genera of intestinal microbiota in patients with AD were distinct from that in controls. Correspondingly, there were 134 genera significantly different in patients with CRC compared with the control (Figure 4A). Within them, there were four significantly different genera among the three groups, namely Acidaminococcus, Alloprevotella, Mycoplasma, and Sphingobacterium (Figure 4B). Notably, the relative abundance of Acidaminococcus was decreased with the order of Control-AD-CRC. We further found that Parvimonas, Peptostreptococcus, Prevotella, Butyricimonas, Alistipes, and Odoribacter were key genera in the network of adenoma-carcinoma sequence (Figure 4C). Furthermore, Random Forest analysis suggested that a combination of the top 10 species showed the best performance in distinguishing AD patients from CRC (AUC = 85.54%, 95% CI: 78.83–92.25%, Figure 4D). Mean Decrease Gini (MDG) and Mean Decrease Accuracy (MDA) coefficients were used to rank the importance of the variables from Random Forest algorithm results (Figures 4E,F). The 10 most important species were *Butyricimonas synergistica*, *Agrobacterium larrymoorei*, *Bacteroides plebeius*, *Lachnospiraceae bacterium feline oral taxon 001*, *Clostridium scindens Clostridium scindens*, *Prevotella heparinolytica, bacterium LD2013*, *Streptococcus mutans*, *Lachnospiraceae bacterium 19gly4*, and *Eubacterium hallii.*

### Association Between Fecal Microbiota and Colorectal Cancer Clinical Characteristics

**FIGURE 5:**
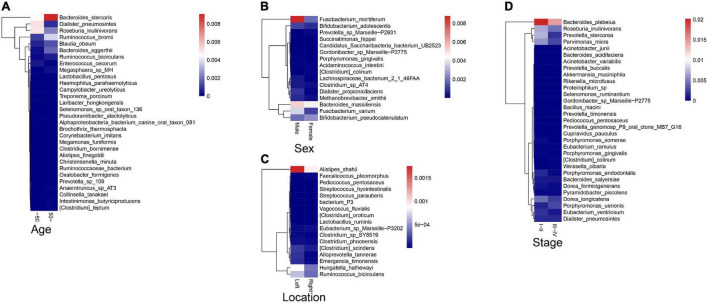
Association between fecal microbiota and CRC clinical characteristics. **(A)** The correlation heatmap between intestinal microbiota and age of patients with CRC; **(B)** The correlation heatmap between intestinal microbiota and gender of patients with CRC; **(C)** The correlation heatmap between intestinal microbiota and the tumor location; **(D)** The correlation heatmap between intestinal microbiota and tumor stage.

To explore the correlations between intestinal microbiota and clinical characteristics of CRC, we created the correlation heatmap according to the different clinicopathologic features of CRC patients. As shown in Figure 5A, 154 patients were divided into two groups according to their ages: 13 CRC patients were younger than 50 years old and 141 patients were older than 50 years old, and 15 bacterial species were higher and another 15 bacterial species were significantly lower in the older patients than that in the younger patients. Stratified by gender, 5 bacterial species increased in female patients including *Prevotella sp. Marseille-P2931, Clostridium colinum*, *Bifidobacterium pseudocatenulatum*, *Gordonibacter sp. Marseille-P2775*, and *Saccharibacteria bacterium UB2523*, while other 11 bacterial species were significantly enriched in male patients (Figure 5B). In addition, the microbiome was different between the patients with the tumor in the left and right colon, in which 17 bacterial species were found obviously changed (7 up and 10 down, left vs. right) (Figure 5C). Furthermore, we compared the relationship between microbiota and tumor stages in CRC (I–II vs. III–IV). The relative abundances of nine bacterial species, including *Porphyromonas uenonis*, *Clostridium colinum*, *Proteiniphilum sp., Selenomonas ruminantium*, *Gordonibacter sp. Marseille-P2775*, *Akkermansia muciniphila*, *Rikenella microfusus*, *Dialister pneumosintes*, and *Weissella cibaria*, were significantly higher in the TNM stage III/IV group than in the TNM stages I/II group (Figure 5D).

## Discussion

In summary, our study identified colorectal adenoma/carcinoma-associated microbes in patients with AD/CRC compared with controls. CRC-associated bacteria altered with the colorectal adenoma-carcinoma sequence. Combining with the ten genera of adenoma-carcinoma sequence associated bacteria can effectively distinguish patients with AD and CRC. The host clinicopathologic features, such as age, sex, tumor location, differentiation degree, and TNM stage, were closely linked to the intestinal microbiome in CRC. Disease outcomes of CRC should account for microbiota characteristics and host factors in the future.

In this study, we found that the species richness and community diversity were not markedly altered between AD and CRC. There was only one family Prevotellaceae that was significantly enriched in CRC compared to AD by LEfSe analysis (data was not shown). The dominant gut microbiota at the genus level in CRC and AD was consistent with the studies reported (14, 17). These results suggest that the differences in microbiota between different studies are consistent, indicating that the differences in microbiota in AD and CRC are less likely to be affected by race, region, diet or others.

Moreover, *Acidaminococcus, Alloprevotella, Mycoplasma*, and *Sphingobacterium* were identified significantly altered among the three groups, in which *Acidaminococcus* was decreased with the order of control-AD-CRC. However, previous study reported that the enrichment of *Acidaminococcus intestini* was found to be higher in carcinomas than in controls and it was negatively correlated with the dietary indices (red meat and serum ferritin) (9). We believe that a single species does not reflect genus differences as a whole, thus leading to inconsistent results. In this study, *Alloprevotella* was enriched in CRC, which was consistent with previous reports on CRC gut microbiota in domestic populations (18). *Alloprevotella* was isolated from the human oral cavity (19), and we also observed the close relationship between colorectal adenoma/carcinoma and oral pathogens, such as *Parvimonas* and *Peptostreptococcus* in the network analysis (20). These bacteria were reported to be enriched in tumor tissues or feces of individuals with CRC or adenomas and involved in the carcinogenesis of CRC (21). Possible mechanisms of pathogenicity include changes in permeability, and interference with signal pathways. Our results provided additional evidence that oral periodontopathic bacteria played an important role in the tumorigenesis of CRC.

Most importantly, our results showed that the combination of the 10 species can distinguish colorectal AD from CRC, which suggested that these 10 special species might be used as potential markers for diagnosing and predicting colorectal AD. Similarly, it was confirmed that CRC-associated bacteria were changed with the degree of malignancy and inflammatory factors (Plasma C-reactive protein and soluble tumor necrosis factor II) increased across the adenoma-carcinoma sequence (20). However, there are still no absolute markers predicting the progression from adenoma to carcinoma. The findings from the current study will contribute to develop the diagnostic biomarkers and therapeutic targets during the progression from adenoma to carcinoma.

Previous studies have shown that CRC-associated microbes varied with adenoma-carcinoma sequence and suggested that the fecal microbiota may contribute to the early diagnosis and treatment of CRC (22). It is known that short-chain fatty acids (SCFAs) producing bacteria is closely related to the occurrence of CRC (23). It may be due to the fact that SCFAs can reduce the expression of inflammatory factors by inhibiting the activation of NF-κB, which can produce anti-inflammatory effects and interfere with precancerous cells in the early stages of CRC development. The data from this study showed that SCFAs producing bacteria, *Eubacterium hallii*, changed in expression as adenoma-carcinoma sequence, which was consistent with results of a previous study. Moreover, a previous study had also reported *Gordonibacter* bacteremia in patients with CRC, indicating that *Gordonibacter sp.* may be related to the occurrence and development of CRC (24). These previous data exemplified can provide objective evidence to support the results of this study.

In addition, previous studies have reported that the composition and relative abundance of intestinal microbiome would be influenced by age, gender, race, or dietary habits (25, 26). Our study has reached similar conclusions. Relative abundance of several bacterial species was altered between different age or sex. Notably, firmicutes dramatically decreased in the old patients compared to the young patients. Consistent with the previous studies, our results also demonstrated that the microbiome was different between the patients with the tumor in the left and right colon (15). The relative abundance of *Bacteroides cellulosilyticus* was significantly increased with the order of differentiation degree in Poor-Moderate-Well and nine CRC-associated bacterial species were significantly higher in TNM stage III/IV group than in TNM stages I/II group. The differences in the microbiota of patients with different tumor location, differentiation degree, and stage provide strong evidence of the tumors–host heterogeneity.

Valuable results were obtained in this study, but there were still deficiencies and limitations. Although this paper revealed adenoma-carcinoma sequence associated intestinal bacteria, it does not provide a more in-depth analysis of the mechanism by which these bacteria participate in the occurrence of the mechanism. In addition, we conducted stratified analysis according to factors such as age, gender, location, and stage in order to further explore the influence of different factors on CRC and intestinal microbiome. However, based on the limitation of samples, the results were unstable. We were well aware of the limitations of results imposed by the lack of sample, and we are actively expanding the sample in order to make the results more reliable and to verify differentially expressed microbiota. Furthermore, this study did not conduct a more in-depth comparison of age subgroup analysis. The 50-year-old distinction did not reflect the differences between different age groups very well, and we will make up for the deficiencies in follow-up research.

In conclusion, several intestinal bacteria changed along the adenoma-carcinoma sequence and might be the potential markers for the diagnosis and treatment of colorectal adenoma/carcinoma. Intestinal microbiota characteristics in CRC should account for the host factors.

## Data Availability Statement

The original contributions presented in the study are included in the article material, further inquiries can be directed to the corresponding authors.

## Ethics Statement

The studies involving human participants were reviewed and approved by the Medical Ethics Committee of The First Affiliated Hospital, Zhejiang University School of Medicine. The patients/participants provided their written informed consent to participate in this study. Written informed consent was obtained from the individual(s) for the publication of any potentially identifiable images or data included in this article.

## Author Contributions

CL proposed the initial idea and designed the experiments. HH conducted the analysis and interpretation of data for the manuscript. XH and YS conducted the procedures. YS supervised and provided consultations during the whole study. YC revised the manuscript. LT solved problems of the knowledge points proposed by the reviewers and revised the final draft of the manuscript. All authors contributed to the article and approved the submitted version.

## Conflict of Interest

The authors declare that the research was conducted in the absence of any commercial or financial relationships that could be construed as a potential conflict of interest.

## Publisher’s Note

All claims expressed in this article are solely those of the authors and do not necessarily represent those of their affiliated organizations, or those of the publisher, the editors and the reviewers. Any product that may be evaluated in this article, or claim that may be made by its manufacturer, is not guaranteed or endorsed by the publisher.

## Footnotes

1
http://qiime.org/scripts/split_libraries_fastq.html

2
http://ccb.jhu.edu/software/FLASH/

3
http://www.drive5.com/usearch/manual/uchime_algo.html

4
https://www.arb-silva.de/

5
http://www.drive5.com/uparse/
